# Meetings of Societies

**Published:** 1920-06

**Authors:** 


### MEETINGS OF SOCIETIES.

### Bristol Medico-Chirurgical Society.

A Clinical Meeting of this Society was held at the University of Bristol on April 14th.

Dr. D. A. Alexander showed a case of Myxœdema ; also a case of Tetany in a boy of 18, with defective physical development and a rather full thyroid gland. Dr. J. M. Fortescue-Brickdale showed a boy of 19 with very defective development, probably due to pancreatic insufficiency. Dr. J. R. Charles showed a case of Myelogenous Leukæmia with symptoms indicative of a lesion of the labyrinth on both sides, coming on simultaneously and acutely. Mr. Hubert Chitty showed a child with an inclusion dermoid of the scalp, and another patient with a lymphangioma of the cheek, under treatment by electrolysis through the mucous membrane. Dr. Richard Clarke brought a specimen of pituitary tumour ; the chief symptoms had been acromegaly, optic neuritis, and diminished glucose tolerance. Mr. J. P. I. Harty and Dr. F. J. A. Mayes showed a case of Epithelioma of Tonsil, Palate, etc., undergoing treatment by diathermy and injections of copper alanin with promising results. Mr. Clifford Moore showed a boy with Schlatter's disease (bilateral). Dr. J. A. Nixon showed a child with morbus cordis and congenital syphilis ; a man with steatorrhœa following gastro-enterostomy (possibly due to implication of the pancreas in the base of a gastric ulcer) ; and a youth convalescent from illness presenting affinities with toxic polyneuritis and also with encephalitis lethargica. Dr. George Parker brought a number of children with infantile diplegia, to whom thyroid extract was being given with benefit ; also cases illustrating paralysis of the serratus magnus and of the trapezius ; and a man with a lesion of the external cutaneous and genitocrural nerves associated with an operation for inguinal hernia. Mr. Rendle Short showed two patients doing well after Albee's operation for tuberculosis of the spine, and a specimen of malignant papilloma of the renal pelvis removed by operation. Dr. J. O. Symes showed two patients exhibiting an indurative erythema of the skin over the calves of the leg. Dr. Watson-Williams showed a patient from whom he had removed a large polypus of the sphenoidal sinus (the polypus also was shown). Dr. Cecil Williams showed a case of œdema of both eyelids in a boy whose mother had been similarly affected after eating oranges ; there was reason to regard the boy's vision as the outcome of a similar " toxic idiopathy." Mr. C. Ferrier Walters showed two patients with Exophthalmic Goitre who had been relieved by the Dunhill operation. Dr. Kenneth Wills showed patients cured by a single dose of X-rays, of (1) rodent ulcer of three years' standing, (2) lupus erythematosus discoides ; also a man with a syphilitic tumour of the maxilla, undergoing successful treatment with novarseno benzol ; and a severe and intractable case of dermatitis herpetiformis. Mr. Duncan Wood showed four ex-service patients, in whom persistent traumatic bony defects had been closed by grafts of living tissue—muscle in three cases and bone in one. Mr. A. J. Wright showed a man with Cicatricial Stenosis of the Larynx following a gun-shot wound, in whom laryngotomy had been performed, to be followed later by a plastic operation for closure by a cartilaginous graft.

